# A tribute to Prof. em. Dr. Dr. h.c. mult. Hildebert Wagner

**DOI:** 10.1080/13880209.2022.2045067

**Published:** 2022-03-10

**Authors:** Rudolf Bauer

**Affiliations:** Institute of Pharmaceutical Sciences, Department of Pharmacognosy, University of Graz, Graz, Austria


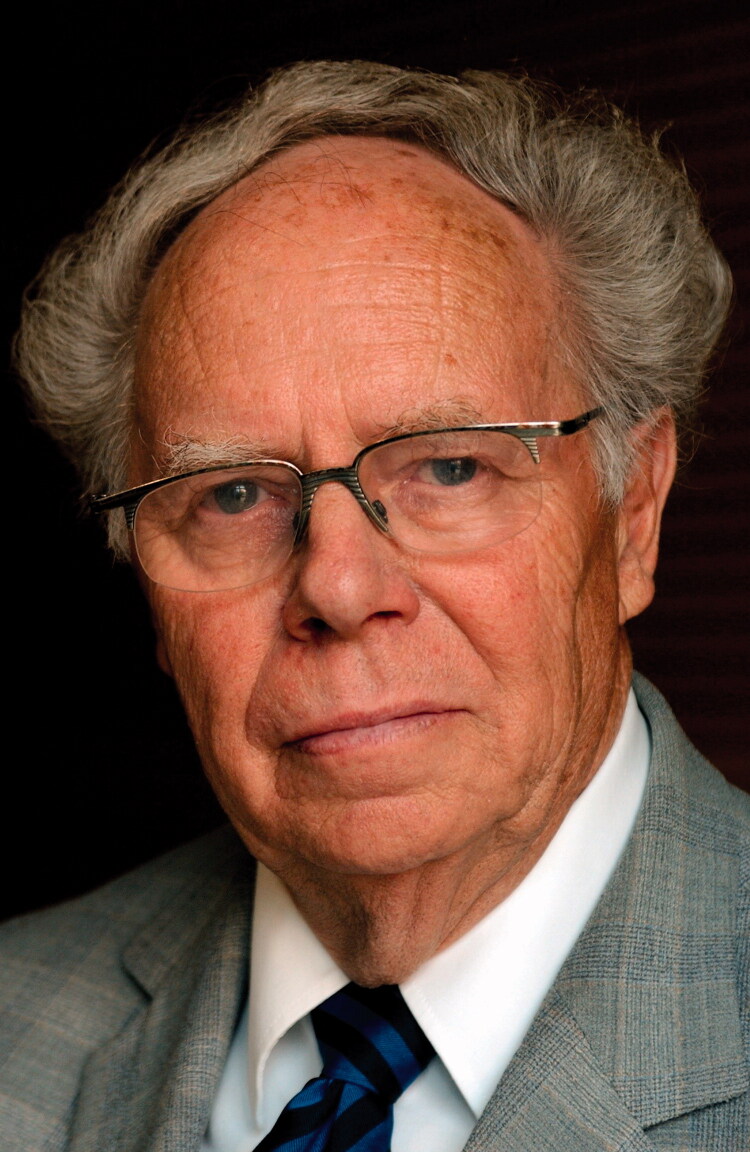


Hildebert Wagner has been my PhD supervisor and one of the major mentors of my scientific career. After a fulfilled scientific life, he passed away on 5^th^ of November 2021 in the age of 92. He has been a world-famous scientist in pharmacognosy. So, it is a good occasion to remember, how he influenced the field and our careers.

As a full professor and as managing director, Hildebert Wagner headed the Institute of Pharmaceutical Biology at University in Munich for over 25 years. There, he also started his pharmaceutical career by studying pharmacy, obtained his PhD under Prof. Ludwig Hörhammer and achieved his habilitation in 1960. He went for a Distinguished Visiting Professorship to Jack Beal at the University of Ohio at Columbus 1970/71.

When I studied pharmacy in Munich in the late 1970s and heard his lectures, I already admired him because of his wisdom and holistic view of medicine. He also differed from the other lecturers because of his appearance: he really looked ‘like a professor’. When I got the chance to do my PhD with him in 1980, I immediately accepted and even quit a position in a pharmacy where I was supposed to do my practical year.

During my doctoral studies, I realised how renown he was in the field, and how well equipped his institute was. Already in the early 1980s, we were regularly using HPLC, MS, NMR and X-ray, and all instruments were available at the institute at that time, which probably made it to one of the best equipped institutes of pharmacognosy in the world. Gerolf Tittel, Otto Seligmann, Mohan Chari, and Hermann Lotter were running these labs at that time. Prof. Wagner was very much interested in structure elucidation of plant constituents and phytochemistry, and had moved pharmacognosy in Munich from a microscopic material science into a modern scientific discipline. Holistic quality control of herbal drugs based on fingerprint analysis was his concept and he was a front-runner in that field shown by his famous books on *Plant Drug Analysis: A Thin Layer Chromatography Atlas* (together with Sabine Bladt and Eva Zgainski), and later *Chromatographic Fingerprint Analysis of Herbal Medicines* (Eds. Hildebert Wagner, Rudolf Bauer, Dieter Melchart, Pei-Gen Xiao, Anton Staudinger). This differentiated him from his predecessor, Ludwig Hörhammer, who was a botanist and published books mainly on microscopic identification. However, Hildebert Wagner was not just interested in the chemical aspects of plants. His final interest was to elucidate their activity. Therefore, he established also a pharmacological lab in his institute, headed at that time by Ksenija Jurcic. This gave us the great chance to do activity guided isolation, and most PhD theses were covering both aspects, constituents and activity of medicinal plants. The discovery of the liver-protective flavonolignans in milk thistly fruits, was certainly one of his major early achievements, and many similar discoveries followed.

He always has been extremely visionary, not afraid to enter new research fields. In the 1990s he started research on polysaccharides and immunomodulation, which was really challenging, because of quite different methods and equipment to use. Echinacea became one of his major research interests and Echinacea polysaccharides were supposed to be produced by biotechnology in commercial scale. Unfortunately, this project failed, but a text book on *Echinacea* remained. In the early 1990s, he strengthened the institute by bringing Meinhard Zenk to Munich, who had been famous in plant tissue culture and biochemical research of plants.

Many colleagues from all over the world visited Prof. Wagner in Munich, gave lectures or did their research sabbatical. Every lecture ended with a post-session in one of the restaurants nearby, and so we also had the chance to meet many of these famous scientists personally, and to start to build our own networks. Every year, there was a peak in lectures during Octoberfest season, because then, the post-session was organised in one of this huge beer halls and the mood always was fantastic. Many of our special guests even were invited to conduct the beer hall brass orchestra. Probably, this was even more exciting to them compared with giving a scientific lecture.

We always had a special relationship to Chicago. Norman Farnsworth has been a very close friend of Hildebert Wagner since he had done his sabbatical in the USA. He, and many colleagues, like Geoffrey Cordell, Harry Fong, John Pezzuto, and Douglas Kinghorn came to Munich frequently. And so, I also established this close relationship to Chicago. I remember very well a visit in the mid-1980s, during which Prof. Wagner recognised the first personal computer at Geoffrey Cordell’s desk, and immediately told me ‘We need that as well!’. During the same trip, Norman Farnsworth had picked us up at the airport and invited us for lunch into Fuddruckers® restaurant offering the World's Greatest Hamburgers®. I hardly managed to bite into this giant burger, but Hildebert Wagner, like a gentleman, took knife and fork, and cut it into small pieces!

A second close collaboration was with colleagues from Hungary. Even before the iron curtain fell down, colleagues from Budapest, like Loránd Farkas, Barbara Vermes, Sandor Antus, Agnes Gottsegen, or András Lipták spent some time in our labs in Munich every year. At lot of papers on synthesis of natural products, especially flavonoids and flavonolignans resulted from these collaborations. Also, my career has been definitively influenced by this Hungarian connection.

In the 1990s, Hildebert Wagner became interested in Traditional Chinese Medicine. As a member of the scientific advisory board, he has been one of the pioneers who contributed to the establishment of the first German TCM hospital in Bad Kötzting. He became responsible for the quality control of all Chinese herbs used in the hospital. At that time, I have been still working in his group in Munich, and immediately he delegated this task to me. So, I came into contact with Chinese medicine and later found out that this is a highly interesting and fascinating field of herbal medicine, so that it became a major research focus of my career. However, Hildebert Wagner’s contacts to China had started even before. Already in the early 1980s, he had visited China and accepted Chinese scientists to work in his laboratories, and he met Xiao Peigen, who became also a very good friend of him, and finally also one of my best mentors in China. It also led to the project ‘*TCM monographs and analysis*’ which we conducted together for many years.

Hildebert Wagner has never been interested to obtain leading functions in scientific societies. He preferred to organise his own network and his own meetings. Nevertheless, the number of international symposia on natural products and phytotherapy he organised in Munich is quite impressive and brought him worldwide recognition. Many colleagues remember a congress dinner in Munich Hofbräuhaus during which Norman Farnsworth showed up with Lederhosen and a Bavarian hat to drink the usual ‘Maß’ (one liter) of beer.

Prof. Wagner published the impressive number of 900 research papers and review articles. It demonstrates not only his outstanding impact into the field, but also his enormous diligence and ambition. He certainly was some kind of a scientific workaholic. In addition, he authored an impressive number of books, just to mention the very well accepted text-book for students *Pharmazeutische Biologie 2* (later co-edited with his Angelika Vollmar and Andreas Bechthold), *Phytotherapie* (together with Markus Wiesenauer), *Evidence and Rational Based Research on Chinese Drugs* (together with Gudrun Ulrich-Merzenich) and *Immunomodulatory Agents from Plants*. It is less known that he also published a book on narcotic drugs (*Rauschgift-Drogen,* Springer Verlag), in which he describes a self-experiment with LSD, in which he perceived his wife as a doll fairy.

Shortly before his retirement, he fulfilled himself a long-cherished wish. In 1994, he founded together with his long-standing friend Norman Farnsworth, *Phytomedicine*, an international journal of phytotherapy and phytopharmacology. In the meantime, edited by Thomas Efferth, it has achieved an impact factor of 5.340 and has become one of the leading international scientific journals in the field. Hildebert Wagner used this journal even long after retirement to propagate his visions of omics-based medicinal plant research and the importance of synergism in the action of medicinal plants.

His whole life has been dedicated to the acknowledgment and the support of phytotherapy. For this goal, he worked tirelessly as a researcher, teacher, and speaker at hundreds of conferences worldwide. He liked travelling, and encouraged me to do the same. He has trained countless young pharmacy and almost a hundred doctoral students, and has accepted many visiting scientists from all over the world. Many of them later obtained leading positions in their countries. So, his impact has really been global and he had friends everywhere. Five of his former doctoral students hold now chairs in the field of pharmaceutical biology.

Many honours and prizes have been awarded to Professor Wagner during his long scientific career. He was honoured by the Society for Medicinal Plant and Natural Product Research (GA) for his outstanding lifetime achievements with the Egon Stahl Medal in Gold, he received the *Norman R. Farnsworth Excellence in Botanical Research Award* of the American Botanical Council, and he has been awarded with the honorary doctorates from the universities of Debrecen, Budapest, Dijon, Helsinki, and Iaci. Moreover, he received the honorary professorships from the universities of Beijing and Arequipa, and has been awarded with honorary memberships of the Hungarian Academy of Science and the American Society of Pharmacognosy.

With him, we have lost not only an excellent scientist, but also a visionary in medicinal plant research. We have to mourn the loss of a pioneering teacher and a valuable academic friend. Our deepest sympathy goes to his wife Ursel and his three children.

## Message from John M. Pezzuto, Editor-in-Chief

On behalf of the Journal, we are extremely grateful to Professor Bauer for providing this remembrance of Professor Wagner. We note other well-deserved tributes have been published as well (Efferth et al. 2021; Kennelly 2021; Scholz and Wessinger 2021; Vollmar, Dirsch et al. 2021a; Vollmar, Dirsch et al. 2021b). In the early 1990s, during the same time Prof. Bauer was at Munich, I too had the good fortune of working with Prof. Wagner, during a sabbatical leave from University of Illinois at Chicago, under the auspices of a fellowship from the Alexander von Humboldt Foundation. During my career, I have been blessed with personally interacting with several legendary icons in our field of natural products, but the impact of working with Prof. Wagner was especially profound. Thinking back to my time in Munich, I was captivated by the extraordinary magnetism of Prof. Wagner. It was as if his institute were a black hole in the universe, which only attracted incredibly talented and dedicated scholars from around the world. Together with his team of professionals, including Peter Wolff, Sabine Bladt, and Ksenija Jurcic during my time, Prof. Wagner created a scientific mecca that catapulted careers, facilitated knowledge and reshaped ideologies. As a small example of international collaborative discovery during my stay, the challenging structure of budmunchiamines (Budapest, Munich, and Chicago) was elucidated (Mar et al. 1991). But more importantly, the indelible influence he had on my life and the lives of so many others is incalculable. We now realise Prof. Wagner’s profound impact as reflected through his multitude of decedents positioned throughout the world. As I departed the institute, in the same vein of John Kennedy’s now famous quote, ‘Ich bin ein Berliner’, the words ‘Ich bin ein Munchener’ rang through my heart and mind. It is comforting to know that Prof. em. Dr. Dr. h.c.mult. Hildebert Wagner received well-deserved recognition and accolades throughout his life and career. It is equally comforting knowing he will always be with us in spirit. Finally, I will reveal one little known fact. As this journal has evolved through the years, the title has been changed a total of four times (Lewandowski and Pezzuto 2012). The last name change, in 1998, to *Pharmaceutical Biology*, was strongly influenced by Hildebert Wagner, who hosted this Editor-in-Chief, at the Institute of Pharmaceutical Biology at the University in Munich.


Univ.-Prof. Dr. DDr.h.c. Rudolf Bauer *Institute of Pharmaceutical Sciences* *Department of Pharmacognosy* *University of Graz* rudolf.bauer@uni-graz.at

